# Persistent Systemic Inflammation is Associated with Poor Clinical Outcomes in COPD: A Novel Phenotype

**DOI:** 10.1371/journal.pone.0037483

**Published:** 2012-05-18

**Authors:** Alvar Agustí, Lisa D. Edwards, Stephen I. Rennard, William MacNee, Ruth Tal-Singer, Bruce E. Miller, Jørgen Vestbo, David A. Lomas, Peter M. A. Calverley, Emiel Wouters, Courtney Crim, Julie C. Yates, Edwin K. Silverman, Harvey O. Coxson, Per Bakke, Ruth J. Mayer, Bartolome Celli, for the Evaluation of COPD Longitudinally to Identify Predictive Surrogate Endpoints (ECLIPSE) Investigators

**Affiliations:** 1 Thorax Institute, Hospital Clinic, Institut d’investigacions Biomèdiques August Pi i Sunyer (IDIBAPS), University of Barcelona and Centro de investigación en red de enfermedades respiratorias (CIBERES), Barcelona, Spain; 2 Fundación Investigación Sanitaria Illes Balears (FISIB), Palma de Mallorca, Spain; 3 GlaxoSmithKline, Research Triangle Park, North Carolina, United States of America; 4 University of Nebraska Medical Center, Omaha, Nebraska, United States of America; 5 University of Edinburgh, Edinburgh, UK; 6 GlaxoSmithKline, King of Prussia, Pennsylvania, United States of America; 7 Respiratory Section, Hvidovre Hospital/University of Copenhagen, Denmark; 8 Manchester Academic Health Sciences Centre, University of Manchester, Manchester, UK; 9 University of Cambridge, Cambridge, UK; 10 University of Liverpool, Liverpool, UK; 11 University of Maastricht, Maastricht, The Netherlands; 12 Brigham and Women’s Hospital and Harvard Medical School, Boston, Massachusetts, United States of America; 13 University of British Columbia, Vancouver, Canada; 14 University of Bergen, Bergen, Norway; Clinica Universidad de Navarra, Spain

## Abstract

**Background:**

Because chronic obstructive pulmonary disease (COPD) is a heterogeneous condition, the identification of specific clinical phenotypes is key to developing more effective therapies. To explore if the persistence of systemic inflammation is associated with poor clinical outcomes in COPD we assessed patients recruited to the well-characterized ECLIPSE cohort (NCT00292552).

**Methods and Findings:**

Six inflammatory biomarkers in peripheral blood (white blood cells (WBC) count and CRP, IL-6, IL-8, fibrinogen and TNF-α levels) were quantified in 1,755 COPD patients, 297 smokers with normal spirometry and 202 non-smoker controls that were followed-up for three years. We found that, at baseline, 30% of COPD patients did not show evidence of systemic inflammation whereas 16% had persistent systemic inflammation. Even though pulmonary abnormalities were similar in these two groups, persistently inflamed patients during follow-up had significantly increased all-cause mortality (13% vs. 2%, p<0.001) and exacerbation frequency (1.5 (1.5) vs. 0.9 (1.1) per year, p<0.001) compared to non-inflamed ones. As a descriptive study our results show associations but do not prove causality. Besides this, the inflammatory response is complex and we studied only a limited panel of biomarkers, albeit they are those investigated by the majority of previous studies and are often and easily measured in clinical practice.

**Conclusions:**

Overall, these results identify a novel systemic inflammatory COPD phenotype that may be the target of specific research and treatment.

## Introduction

Non Communicable Diseases (NCDs), including cardiovascular diseases, chronic respiratory diseases, cancer and diabetes, are the major global health problem of the century [1]. They are the world leading cause of disease burden and mortality, are increasing in prevalence even in low- and middle-income countries, the costs incurred by uncontrolled NCDs are substantial, and they are an under-appreciated cause of poverty and hinder economic development [2]. Chronic obstructive pulmonary disease (COPD) is the major respiratory NCD [2], [3]. It affects around 10% of the adult population [4], and it is predicted that it will be the third cause of death and disability in the world by the year 2020 [5].

Persistent, low-level, systemic inflammation is thought to play a significant pathogenic role in many NCDs including COPD [6]. Elevated circulating levels of white blood cells (WBC), C-reactive protein (CRP), interleukins 6 (IL-6) and 8 (IL-8), fibrinogen and tumor necrosis factor alpha (TNFα) have been reported in patients with COPD [7]–[9]. However, most previous studies were small and cross-sectional, showed large variability between patients, did not consider the effects of potential confounders, such as smoking status and treatment with anti-inflammatory agents and, importantly, did not investigate their relationship with relevant clinical outcomes of the disease.

The inflammatory response is a complex network of many different cells and molecules [10], [11]. Addressing this complexity is a key challenge for a better understanding and treatment of NCDs in general [2], and COPD in particular [12], [13]. The emerging field of network medicine provides a platform to explore the complexity of apparently distinct phenotypes of a disease [14].

Because COPD is a complex disease with pulmonary and extra-pulmonary manifestations [15], the identification and prospective validation of specific clinical phenotypes is key for the development of novel and more effective therapies [16]. We hypothesized that the persistence of systemic inflammation in COPD constitutes a novel COPD phenotype [16] because it does not occur in all COPD patients but, when persistently present, it is associated with worse clinical outcomes. To test this hypothesis, we determined in 1755 COPD patients, 297 smokers and 202 non-smoker controls included in the ECLIPSE study [17]: *(1)* the prevalence, temporal stability and network pattern (inflammome [18]) of the six inflammatory biomarkers most often studied in COPD (WBC count, CRP, IL-6, IL-8, fibrinogen and TNFα) [8], [9]; and, *(2)* their relationship with clinical characteristics and relevant outcomes at 3 years follow-up. Our results support that the presence of persistent systemic inflammation constitutes a novel COPD phenotype.

## Methods

### Study Design and Ethics

The design and methods of the ECLIPSE study (Clinicaltrials.gov identifier NCT00292552; GSK study code SCO104960) have been published previously [17]. Briefly, ECLIPSE is an observational, longitudinal study in which, after the baseline visit, participants are evaluated at 3 months, 6 months and then every 6 months for 3 years. ECLIPSE complies with the Declaration of Helsinki and Good Clinical Practice Guidelines, and has been approved by the ethics committees/institutional review boards of the participating centers (listed in Information S1). All participants provided written informed consent.

### Population

We recruited into the ECLIPSE study 2164 patients with COPD, 337 smoking and 245 non-smoking controls [15]. COPD patients were male/female subjects aged 40–75 yrs., with a baseline post-bronchodilator Forced Expiratory Volume in 1 sec. (FEV_1_) <80% of the reference value, an FEV_1_/Forced Vital Capacity (FVC) ratio ≤0.7 and a current or former smoking history of ≥10 pack-yrs., who did not report a COPD exacerbation within the 4 weeks that preceded enrollment [17]. Controls were healthy male/female subjects aged 40–75 yrs. with normal spirometry; smoker controls were current or ex-smokers with a smoking history ≥10 pack-yrs. whereas nonsmoking controls had a smoking history of <1 pack-yrs. In the current analysis we included only those subjects with complete data for the six biomarkers analyzed (1755 COPD patients (81% of the COPD patients recruited into ECLIPSE), 297 smokers with normal lung function (88%) and 202 non-smokers (83%)).

### Measurements

The methodology used in the ECLIPSE study has been published at length elsewhere [15], [17]. Briefly, validated questionnaires were used to record clinical data and nutritional status was assessed as the body mass index (BMI) and fat-free mass index (FFMI), the latter measured by bioelectrical impedance [15], [17]. Exacerbations in the year prior to the study and during follow up were recorded as reported elsewhere [19]. Spirometry and the 6 minute walking distance (6MWD) were performed according to international guidelines [20], [21]. The European Community for Coal and Steel Spirometric reference values were used [22]. The BODE index was calculated as previously described [23]. Low-dose computed tomography (CT) scan of the chest (GE Healthcare or Siemens Healthcare) [15], [17] was obtained; the percentage of lung CT voxels <−950 Hounsfield Units was used to quantify level of emphysema (Pulmonary Workstation 2.0. VIDA Diagnostics, Iowa City, IA, USA) [24].

Of particular interest for the current study are the biomarker measurements. To this end, peripheral venous blood was collected into Vacutainer tubes, in the morning, after fasting overnight, at baseline and at the one year follow-up visit. Circulating WBC count was measured in a central clinical laboratory. Serum was prepared by centrifugation of whole blood at 1500 g for 10 to15 minutes and plasma (EDTA as the anticoagulant) was obtained by centrifugation at 2000 g for 10 to 15 minutes. Samples were stored at –80° until analyzed centrally. IL-6, IL-8 and TNF-α serum concentrations were determined by validated immunoassays (SearchLight Array Technology, Thermo Fisher Scientific, Rockford, IL, USA), whereas CRP (Roche Diagnostics, Mannheim, Germany) and fibrinogen (K-ASSAY fibrinogen test, Kamiya Biomedical Co., Seattle, WA, USA) levels were measured using immunoturbidometric assays validated for use with EDTA plasma. The lower limit of quantification (LLQ) for IL-6, IL-8, TNF-α, CRP and fibrinogen were 0.4 pg/mL, 0.8 pg/mL, 4.7 pg/mL, 0.02 µg/mL, and 5.4 mg/dL, respectively. Biomarker concentrations were below the LLQ in some individuals. To avoid a downward bias of the population data, a nominal level of half of the LLQ value was used in the analysis in individuals with values below the LLQ [25].

### Statistical Analysis

Results are shown as mean (SD), median values [interquartile range [IQR]], frequency distribution (quartiles) or proportions, as appropriate. Because none of the continuous variables were normally distributed, Kruskal-Wallis tests were used to analyze the statistical significance of differences between groups. Differences in categorical variables were assessed using Cochran-Mantel-Haenszel tests. Logistic regression was used to investigate factors contributing to persistent systemic inflammation in patients with COPD. P-values less than 0.05 (two sided) were considered significant.

## Results

### Demographics and Clinical Data

Table 1 presents the main demographic and clinical characteristics of all participants at recruitment. On average, COPD patients had moderate to severe airflow limitation and, as expected, complained of more symptoms, exacerbations and cardiovascular disease than controls. Non-smokers and smokers without COPD had normal spirometry and were slightly younger than the COPD patients. There were a higher proportion of females among controls.

**Table 1:** Mean (SD), median [IQR], or proportion of the main characteristics of the three groups of participants at baseline.

				P-values			
	COPD Subjects (N = 1755)	Smoker Controls (N = 297)	Non-smokerControls (N = 202)	Overall	COPD Subjects vs Smoker Controls	COPD Subjects vs Non-smoker Controls	Smoker Controls vs Non-smoker Controls
**Demographics**							
Age (yrs.)	63.5 (7.1)	55.5 (8.8)	53.0 (8.6)	<0.001	<0.001	<0.001	0.002
Male (%)	1160 (66%)	162 (55%)	76 (38%)	<0.001	<0.001	<0.001	<0.001
Current smoker (%)	640 (36%)	187 (63%)	0	<0.001	<0.001	<0.001	<0.001
Smoking, pack-years	48.9 (27.1)	31.7 (22.1)	0.2 (1.2)	<0.001	<0.001	<0.001	<0.001
BMI, kg/m^2^	26.5 (5.6)	26.7 (4.6)	27.7 (5.5)	0.017	NS	0.006	NS
FFMI, kg/m^2^	17.2 (2.9)	17.0 (2.6)	17.3 (2.7)	NS	NS	NS	NS
Chronic bronchitis (%)	599 (34%)	29 (10%)	3 (1%)	<0.001	<0.001	<0.001	<0.001
mMRC Score	1.7 (1.1)	0.2 (0.5)	0.1 (0.4)	<0.001	<0.001	<0.001	0.001
SGRQ-C Total Score	49.6 (20.1)	9.4 (11.9)	5.0 (6.7)	<0.001	<0.001	<0.001	<0.001
Exacerbation rate (Prior Year)	0.8 (1.2)	0.0 (0.0)	0.0 (0.0)				
ICS Use (%)	1253 (71%)	3 (1%)	0	<0.001	<0.001	<0.001	NS
Cardiovascular disease (%)	577 (33%)	45 (15%)	31 (15%)	<0.001	<0.001	<0.001	NS
Statin Use (%)	396 (23%)	48 (16%)	25 (12%)	<0.001	0.013	<0.001	NS
**Physiology and Imaging**							
FEV_1_/FVC, %	44.6 (11.4)	79.1 (5.1)	81.4 (5.2)	<0.001	<0.001	<0.001	<0.001
FEV_1_ (L)	1.35 (0.52)	3.31 (0.75)	3.34 (0.79)	<0.001	<0.001	<0.001	NS
FEV_1%_ Predicted	48.2 (15.6)	108.8 (12.1)	115.3 (14.2)	<0.001	<0.001	<0.001	<0.001
FEV_1_ reversibility, %	10.9 (13.8)	4.4 (5.9)	2.6 (4.0)	<0.001	<0.001	<0.001	<0.001
6MWD, m	371 (121)						
BODE Index	3.1 (2.1)						
%LAA on CT (<−950HU)	17.6 (12.2)	2.4 (3.1)	3.9 (3.9)	<0.001	<0.001	<0.001	<0.001
**Inflammatory Biomarkers**							
White Blood Cells (X10^6^/ml)	7.6 [6.3,9.0]	7.1 [6.1,8.6]	5.8 [5.0,7.0]	<0.001	<0.001	<0.001	<0.001
High Sensitivity CRP (mg/l)	3.2 [1.5,7.1]	1.6 [0.8,3.3]	1.3 [0.6,2.7]	<0.001	<0.001	<0.001	0.041
IL-6 (pg/ml)	1.5 [0.8,3.1]	0.6 [0.3,1.3]	0.4 [0.2,0.9]	<0.001	<0.001	<0.001	<0.001
IL-8 (pg/ml)	6.9 [3.2,13.3]	7.8 [3.8,14.2]	4.3 [2.3,7.2]	<0.001	0.013	<0.001	<0.001
Fibrinogen (mg/dl)	448.0 [388.0,517.0]	391.0 [348.0,436.0]	369.0 [326.0,432.0]	<0.001	<0.001	<0.001	0.003
TNF-alpha (pg/ml)	2.35 [2.35,7.80]	2.35 [2.35,40.70]	2.35 [2.35,2.35]	<0.001	<0.001	<0.001	<0.001

NS: non-significant.

### Cross-sectional Analysis of Systemic Inflammation at Recruitment

**Figure 1:**
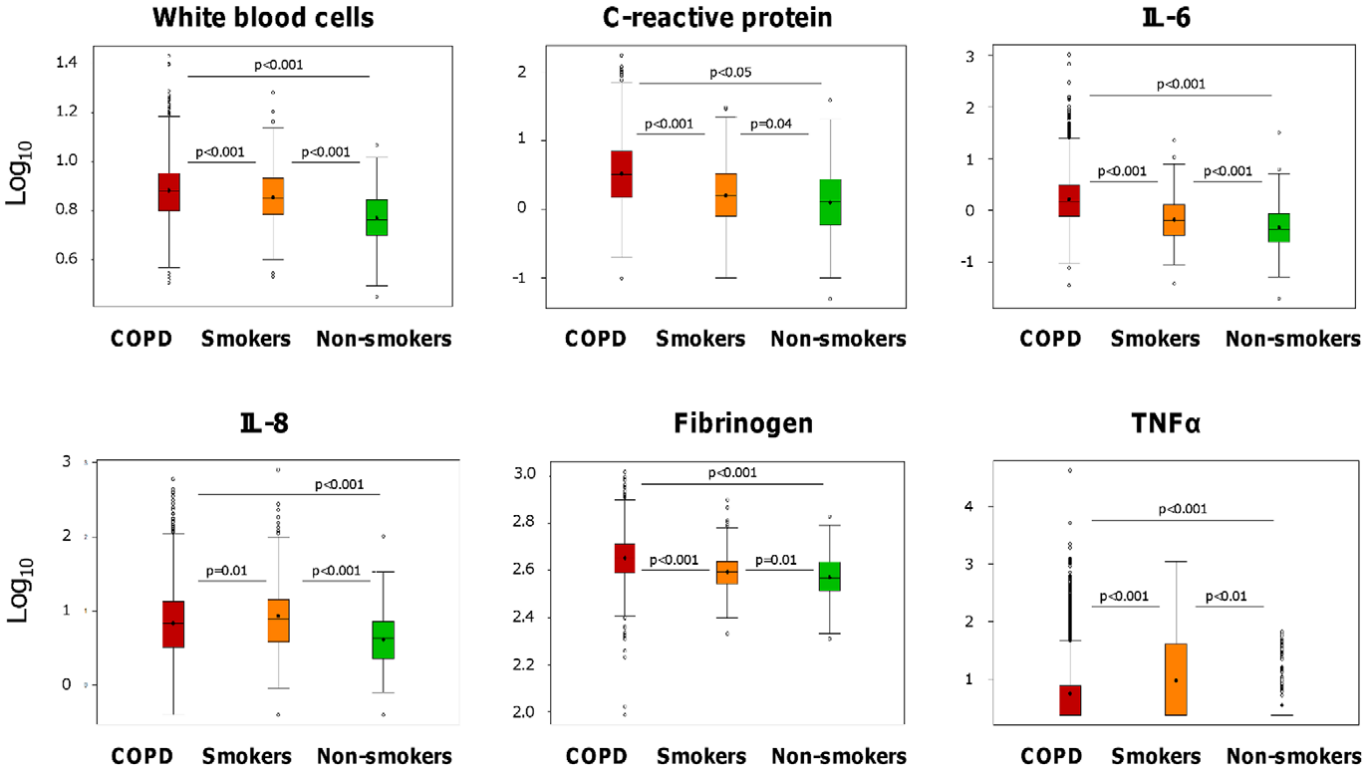
Box plot (log scale) of the different biomarkers determined at baseline in COPD patients, smokers with normal lung function and nonsmokers. For further explanations, see text.

Figure 1 shows a box plot of the six inflammatory biomarkers measured at recruitment in the three groups of subjects studied, and Table 1 shows their median [IQR] values. Despite large variability within each group (note the logarithmic scale on Figure 1) and relatively small absolute differences between groups (Table 1), on average the WBC count and levels of CRP, IL-6 and fibrinogen were significantly higher in COPD patients than in smokers with normal lung function and nonsmokers, whereas IL-8 and TNFα values were higher in smokers without COPD (Figure 1, Table 1). CRP, IL-6 and fibrinogen were not influenced by active smoking, and WBC counts were only slightly higher in current smokers compared with former smokers and non-smokers (Table S1). In patients with COPD, the WBC count and the serum levels of CRP, IL-6 and fibrinogen, but not those of IL-8 and TNFα, tended to increase with the severity of airflow limitation (Table S2).In absolute terms, differences in the levels of systemic biomarkers between GOLD stages were small and often not consistent between stages (Table S2).

To determine the prevalence of elevated inflammatory biomarkers, values >95^th^ percentile of healthy non-smokers were considered abnormal [26], [27] (Table S3). Seventy seven percent of non-smokers, 42% of smokers and, importantly, 30% of COPD patients did not have any abnormal biomarker, so defined. Figure S1 shows that the percentage of individuals with abnormal biomarker values was significantly shifted towards the right (more inflammation) in smokers (vs. nonsmokers), and more so in patients with COPD (vs. smokers and nonsmokers).

**Figure 2:**
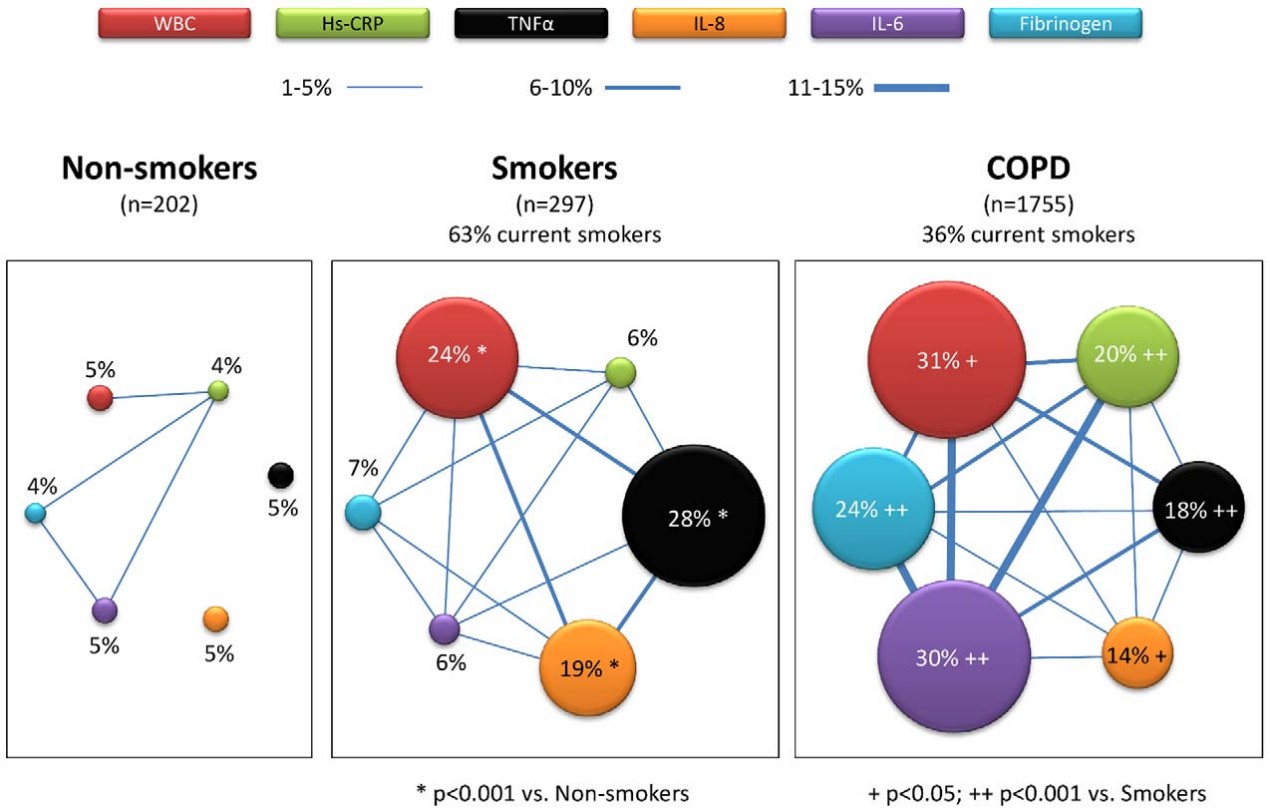
Network layout of the systemic inflammatory response (inflammome) in non-smokers (n = 202), smokers with normal lung function (n = 297) and COPD patients (n = 1755) at recruitment. Each node of the network corresponds to one of the six inflammatory biomarkers determined in this study (see color code), and its size is proportional to the prevalence of abnormal values (>95^th^ percentile of non-smokers) of that particular biomarker in that particular group of subjects (precise figure shown inside of each node). Two nodes are linked if more than 1% of subjects in the network share abnormal values of these two biomarkers, its width being proportional to that proportion. For further explanations, see text.

Figure 2 presents a network layout of the systemic inflammatory pattern in the three groups of participants. Each node of the network represents one biomarker, its size being proportional to the percentage of abnormal values (exact figure shown inside) in each group. Nodes are linked if 1% or more of subjects share abnormal values for the particular biomarkers, and the width of the link represents the size of this percentage. In non-smokers, nodes are, by definition, small but, interestingly, links are rare and thin, indicating the virtual absence of an inflammome (Figure 2). In smokers with normal spirometry, some nodes (WBC, IL-8 and TNFα) are larger (p<0.001) than in nonsmokers whereas others (CRP, IL-6 and fibrinogen) have a similar size (p = ns), and a network (inflammome) is now clearly visible, with many thick linking lines (Figure 2). In patients with COPD, the network is further developed (more and thicker links) with some nodes (WBC (p<0.03), CRP (p<0.001), IL-6(p<0.001) and fibrinogen (p<0.001)) increasing, and others (IL-8 (p<0.02) and TNFα (p<0.001)) decreasing in size as compared with smokers with normal lung function (Figure 2). This pattern was maintained when current smokers with normal spirometry were compared with former smokers with COPD (Figure S2). Because IL-8 and TNFα appear to be primarily markers of smoking and not of COPD (Table S1, and Figures 2 and S2), we excluded them from further analysis.

### Longitudinal Stability of Systemic Inflammation

**Figure 3:**
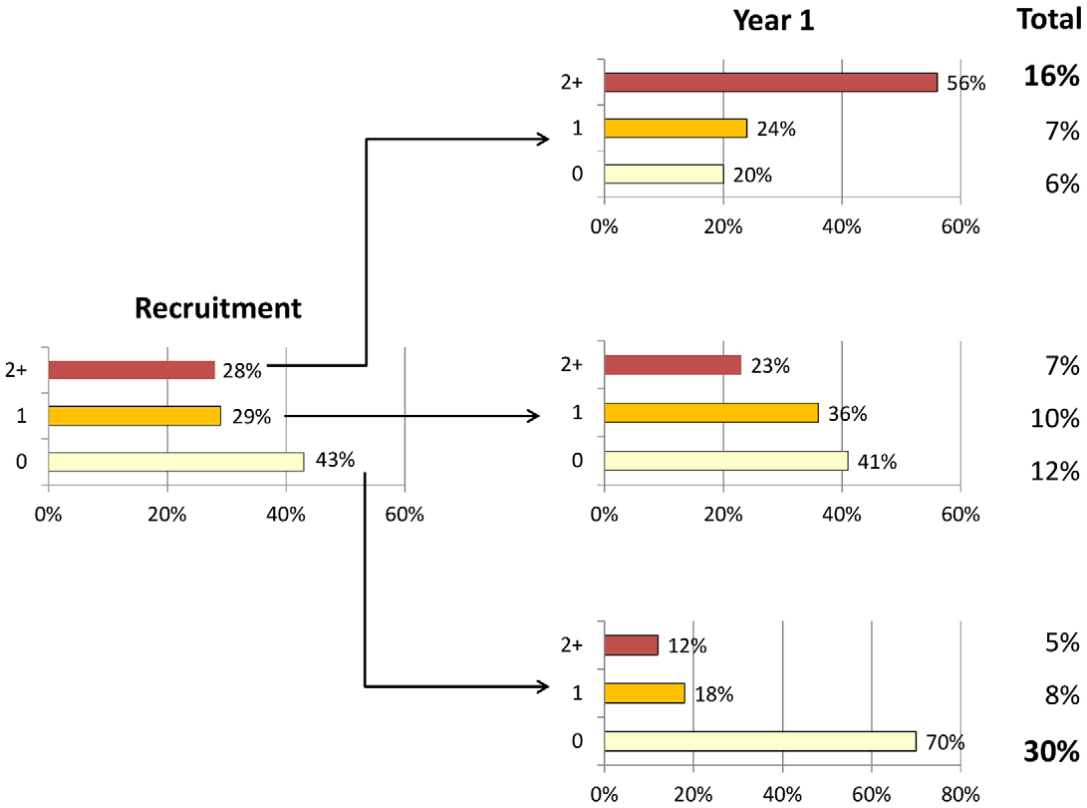
Proportion of patients with none, one, or two (or more) biomarkers (WBC count, CRP, IL-6 and fibrinogen) in the upper quartile of the COPD distribution, at baseline (left bars) and after one year follow up (right bars). For further explanations, see text.

Figure 3 shows the proportion of COPD patients with zero, one and two (or more) biomarkers (WBC, CRP, IL-6 and fibrinogen) in their upper quartile distribution determined at baseline and one year later (Table S4). At recruitment (left bars), 28% of the COPD patients had two or more biomarkers in the upper quartile, and this was still the case for 56% of these individuals one year later (right-top bars). Overall, subjects with 2 or more biomarkers in the upper quartile both at baseline and after one year represent 16% of the population of patients studied (Figure 3).In contrast, 43% of COPD patients did not have any biomarker in the upper quartile of their distribution and this remained true for 70% of these subjects one year later (right-bottom bars). These subjects correspond to 30% of the total population studied. Their proportion decreased with the GOLD stage of airflow limitation whereas that of persistently inflamed patients increased slightly (Figure S3).The systemic inflammome determined at baseline was stable for the four biomarkers analyzed at one year follow-up in each group of participants (Figure S4).

### Relationship between Systemic Inflammation, Disease Characteristics and Clinical Outcomes

Table 2 compares the baseline demographics, clinical, functional and imaging characteristics of the patients with (2+ elevated biomarker levels) and without (none) persistent (at baseline and 1 year later) systemic inflammation. Age and gender were similar in both groups, but patients with persistent systemic inflammation were more obese, had slightly more cumulative exposure to smoking and were more likely to be current smokers, were more symptomatic, had worse health status, reported a higher prevalence of COPD exacerbations and cardiovascular disease and a higher proportion used inhaled steroids, but not statins. Airflow limitation was slightly worse in these patients, as were their exercise tolerance and BODE index, but neither the prevalence of chronic bronchitis, nor the degree of airflow limitation reversibility or the extent of CT- emphysema were different between the two groups (Table 2). Table 3 presents the results of the logistic regression analysis for persistent systemic inflammation in COPD. Age, BMI (but not FFMI, suggesting a role for adipose tissue), current smoking, health status and airflow limitation were associated with increased risk of persistent, systemic inflammation. Interestingly, gender, cumulative smoking exposure, presence of chronic bronchitis, prior exacerbation rate, use of ICS, history of cardiovascular disease, statin use, exercise tolerance and the presence of emphysema were not associated with the presence of persistent systemic inflammation in COPD (Table 3).

**Table 2:** Comparison of baseline demographics, clinical, physiological and imaging characteristics of COPD patients with none or two (or more) biomarker levels in the upper quartile of the COPD distribution both at baseline and at one year follow-up.

	Number of Biomarkers Elevated at Both Visits		
	0, N = 431 (30%)	2+, N = 220 (16%)	p-value
**Demographics and clinical data**			
Age (yrs.)	63.2 (6.9)	64.5 (6.5)	0.03
Male (%)	276 (64%)	152 (69%)	NS
BMI, kg/m∧2	25.6 (4.8)	29.4 (7.3)	<0.001
FFMI, kg/m∧2	16.9 (2.6)	18.3 (3.9)	<0.001
Smoking, pack-years	44.2 (24.2)	54.7 (31.6)	<0.001
Current smoker (%)	133 (31%)	88 (40%)	0.02
Chronic Bronchitis (%)	123 (29%)	78 (35%)	NS
mMRC Score	1.3 (0.9)	2.0 (1.1)	<0.001
SGRQ-C Total Score	42.3 (19.1)	56.8 (19.8)	<0.001
Exacerbation rate (Prior Year)	0.7 (1.2)	1.1 (1.5)	<0.001
ICS Use (%)	284 (66%)	174 (79%)	<0.001
Cardiovascular disease (%)	113 (26%)	80 (36%)	0.007
Statin Use (%)	96 (22%)	56 (25%)	NS
**Physiology and Imaging**			
FEV_1_ (L)	1.49 (0.53)	1.26 (0.44)	<0.001
FEV_1%_ Predicted	52.6 (15.2)	46.0 (14.5)	<0.001
FEV_1_ reversibility, %	11.4 (14.8)	11.7 (13.7)	NS
FEV_1_/FVC, %	46.1 (11.1)	44.5 (10.9)	NS
6MWD, m	419 (109)	336 (117)	<0.001
BODE Index	2.3 (1.8)	3.8 (2.0)	<0.001
%LAA on CT (<−950HU)	17.3 (12.4)	16.8 (10.1)	NS

NS: non-significant.

**Table 3:** Summary of logistic regression for persistent systemic inflammation (defined as in upper quartile at both visits for at least 2 biomarkers).

	Odds Ratio (95% CI)	p-value	AUC for Model
			0.76
**Demographics and clinical data**			
**Age (yrs.)**	**1.045 (1.014, 1.077)**	**0.004**	
Female vs. Male	0.645 (0.383, 1.085)	0.098	
**BMI, kg/m^2^**	**1.125 (1.063, 1.190)**	**<0.001**	
Fat free mass index, kg/m^2^	0.979 (0.866, 1.106)	0.728	
**Current smoker vs. Former smoker**	**2.228 (1.471, 3.375)**	**<0.001**	
Smoking, pack-years	1.004 (0.997, 1.011)	0.217	
Chronic bronchitis	0.929 (0.624, 1.384)	0.717	
mMRC Dyspnea Score	0.949 (0.756, 1.191)	0.65	
**SGRQ-C Total Score**	**1.017 (1.004, 1.030)**	**0.012**	
Exacerbation rate (prior year)	1.097 (0.956, 1.260)	0.187	
ICS Use	1.354 (0.850, 2.159)	0.202	
Cardiovascular disease	0.714 (0.470, 1.085)	0.114	
Statin Use	0.899 (0.576, 1.403)	0.638	
**Physiology and imaging**			
**FEV1% Predicted**	**0.975 (0.956, 0.995)**	**0.014**	
FEV1 Reversibility	0.999 (0.987, 1.011)	0.841	
FEV1/FVC (%)	1.000 (0.972, 1.028)	0.977	
6MWD (m)	0.998 (0.997, 1.000)	0.13	
%LAA	0.987 (0.965, 1.008)	0.219	

Statistically significant factors are highlighted in bold. For further explanations, see text.

During the three year follow up, both all-cause mortality (13% vs. 2%, p<0.001) and the annual rate of COPD exacerbations (adjusted for prior exacerbation rate (1.5 (1.5) vs. 0.9 (1.1), p<0.001) [19]) were significantly higher in individuals with persistent systemic inflammation compared with those without it. By contrast, neither the rate of FEV_1_ decline (−33±46 vs. −33±43 ml/yr., p = 0.905), weight loss (−1.3 (6.7) vs. −0.7 (5.5) Kg, p = 0.504) or the occurrence of new cardiovascular events (7% vs. 9%, p = 0.500) were significantly different between these two groups.

## Discussion

This study provides three relevant and novel observations. First, it characterizes the systemic inflammatory network pattern (inflammome) in patients with COPD and distinguishes it from that of smokers with normal lung function and non-smokers. Secondly, it shows that systemic inflammation is not a constant feature in all COPD patients, since about a third of those studied here did not have any abnormal biomarker at baseline and about the same proportion remained ‘non-inflamed’ after one year of follow up. Finally, it identifies a subgroup of COPD patients with persistently elevated inflammatory biomarker levels that, despite relatively similar lung function impairment, had significantly increased all-cause mortality and exacerbation frequency. These inflamed patients may therefore constitute a novel distinct phenotype within the larger group of patients with COPD and could be the target of novel therapeutic strategies.

Several studies have previously reported elevated levels of circulating WBC, CRP, IL-6, IL-8, fibrinogen and TNFα in patients with clinically stable COPD [8], [28]–[37]. Yet, they were limited because of the relatively small numbers of patients studied, the large variability of values observed, the fact that measurements were mostly made on a single occasion, potential confounders such as smoking status and treatment with anti-inflammatory drugs were not considered and, importantly, the longitudinal relationship with relevant clinical outcomes of the disease could not be established because of their cross-sectional design. Our study overcomes these limitations and provides, therefore, novel information on the true prevalence of systemic inflammation in COPD and its importance in the progression of disease.

The inflammatory response is a complex network of multiple cell types and mediators [10], [11] which the emerging field of network medicine is only beginning to decipher [14], [38]. We used this approach [2], [12], [13]to identify relationships between systemic inflammatory biomarkers (the inflammome) [18] among smokers with and without COPD. We recognize that our results are incomplete but they showed that, at variance with current understanding [7]–[9], systemic inflammation is not a constant feature of COPD and that, when present for at least 1 year, it is associated with worse COPD outcomes at 3 years follow-up. Age, gender and smoking exposure were similar between non-inflamed and inflamed patients but the latter were more obese, dyspneic, had lower health related quality of life, more frequent exacerbations, worse exercise tolerance, a higher BODE index and reported more cardiovascular disease, despite similar use of statins (Table 2). Interestingly, although airflow limitation was slightly worse in patients with persistent inflammation, most pulmonary characteristics of COPD, such as the prevalence of chronic bronchitis, the degree of emphysema, the bronchodilator response and the rate of FEV_1_ decline during follow-up, were similar in both groups (Table 2). Logistic regression analysis identified age, BMI, current smoking, health status and airflow limitation as risk factors for persistent inflammation whereas gender, cumulative smoking exposure, presence of chronic bronchitis, prior exacerbation rate, use of ICS, history of cardiovascular disease, statin use, exercise tolerance and the presence of emphysema were excluded (Table 3). Taken together, these observations suggest that systemic inflammation in COPD need not parallel the severity of the lung disease and raises questions about its pulmonary origin (the “spill-over” hypothesis) [9]. In contrast, the fact that persistently inflamed patients were more obese supports a potential systemic origin of inflammation [39], although other potential mechanisms, such as the presence of airway bacterial colonization [40] and/or sleep apnea syndrome overlap [41] cannot be excluded because they were not investigated in ECLIPSE. The origin of systemic inflammation in COPD remains to be determined. However, our findings are consistent with those of Garcia-Aymerich *et al*, who using a different methodological approach (cluster analysis) also identified a “systemic” COPD subtype characterized by more systemic inflammation and a higher proportion of obesity in 342 COPD patients followed during 4 years [42].

An important observation of our study is that all-cause mortality (13% vs. 2%) and the annual rate of moderate/severe COPD exacerbations (1.5 vs. 0.9 per year) during the 3 year follow-up were higher (p<0.001) in the persistently inflamed patients, compared with non-inflamed patients. These observations are clinically relevant because the severity of airflow limitation has been used so far as the most important criteria to guide therapy in COPD [43], whereas our study shows that patients with similar levels of airflow limitation may have different outcomes depending on the presence or absence of persistent systemic inflammation. Indeed, a persistent elevation of systemic inflammatory biomarkers can occur even in patients with moderate airflow limitation (Figure S3). In this context, it is worth noting that among the 220 patients identified in this study with persistent systemic inflammation (Table 2), 89 (40%) were frequent exacerbators according to the definition of Hurst *et al* [19], an additional 61 (28%) had a single exacerbation, and the remaining 70 (32%) reported no exacerbations during the first year of follow up, suggesting that the frequent exacerbator phenotype [19] and the persistently inflamed phenotype described here are not necessarily identifying the same individuals. Finally, given the limited efficacy of inhaled corticosteroids in reducing systemic inflammation in COPD [44], patients with persistent systemic inflammation may require a different therapeutic approach for the optimal management of their disease that will have to be explored in future studies.

Our study has several strengths and limitations. To date, it provides the largest longitudinal investigation of systemic inflammatory biomarkers in a group of stable, well characterized COPD patients and compares their results to those of smoking and non-smoking controls [17]. This latter aspect proved important for the proper interpretation of the findings reported here, since the large biomarker variability observed required the establishment of upper normal values. Likewise, given the significant effect of smoking identified, any accurate interpretation of abnormal levels of inflammatory markers in COPD must take it into account. The fact that patients were followed prospectively for 3 years is another strength of our study because it not only allowed the assessment of the temporal stability of the biomarker levels but, importantly, the investigation of their relationship with clinically relevant outcomes, and thus the identification of a distinct subgroup of COPD patients with worse clinical outcomes associated with the persistence of systemic inflammation. Our study also has some potential limitations. First, this is a descriptive study, so our results only show associations and do not prove causality. Besides, since this is an exploratory analysis, we opted to identify as many possible differences for further investigation by not adjusting for multiple comparisons. Hence, our analyses and conclusions will need to be replicated either prospectively in a study powered for these hypotheses or in other cohorts that contain similar data. Second, the biology of the inflammatory response is complex and we studied only a limited panel of biomarkers. However, the biomarkers we chose correspond to those investigated by the majority of previous studies [8], [28]–[33] and are often and easily measured in clinical practice. Yet, we did not study markers of tissue repair, and it is likely that the balance between inflammation and repair is important for the pathobiology of COPD [45]. Third, patients were recruited into ECLIPSE mostly from hospital clinics and were treated according to their local physician. These considerations need to be taken into account when comparing results with untreated patients or patients managed in primary care since no patients with mild airflow limitation (GOLD grade 1) were included in the study. Finally, mortality data refers to all-cause mortality since cause-specific mortality was not recorded in the study.

In conclusion, this study begins to describe the systemic inflammatory network pattern (inflammome) associated with COPD and how it differs from that of smokers with normal lung function. It also identifies a sub-group of COPD patients with persistently increased biomarkers levels that is associated with a higher incidence of exacerbations and worse survival despite similar lung impairment, suggesting that this constitutes a novel COPD phenotype [16]. Future clinical trials will have to determine the best therapeutic strategy for these patients. This may have important therapeutic implications also for other major non-communicable diseases, including cardiovascular and metabolic diseases, also characterized by chronic low-level systemic inflammation [7], [46].

## Supporting Information

Figure S1 **Frequency distribution of the percentage of individuals in each group with none, one or more abnormal biomarker values (>95^th^ percentile of the nonsmoker controls) at baseline.** For further explanations, see text.
(TIF)

Figure S2 **Systemic inflammome of non-smokers (n = 202), current smokers (only) with normal lung function (n = 187) and former-smokers (only) with COPD (n = 1115) at baseline.** IL-8 and TNFα are very much influenced by current smoking whereas hs-CRP, IL-6 and fibrinogen are COPD-related inflammatory biomarkers. WBC counts are influenced both by smoking and COPD. For further explanations, see text.
(TIF)

Figure S3 **Percentage of COPD patients, by GOLD stage of airflow limitation severity, with none (blue bars) or 2+ biomarkers (red bars) in the upper quartile of the COPD distribution of values both at baseline and after one year follow-up.** For further discussion, see text.
(TIF)

Figure S4 **Systemic inflammome of the four biomarkers analyzed at baseline (upper panels) and at one year follow-up (bottom panels) in the same individuals in each group (note the same n value).** Differences between groups were maintained after one year follow-up but were basically non-existent within groups, indicating stability of the systemic inflammome in each group. For further explanations, see text.
(TIF)

Table S1 **Median [IQR] of the inflammatory biomarkers determined at baseline in COPD patients and smokers with normal lung function by smoking status.**
(DOCX)

Table S2 **Median [IQR] of the inflammatory biomarkers determined at baseline in COPD patients by GOLD stages of airflow limitation.**
(DOCX)

Table S3 **95^th^ percentile values of the six biomarkers determined in healthy non-smokers at baseline.** For further explanations, see text.
(DOCX)

Table S4 **Summary of 75^th^ percentile value of the four biomarkers determined in COPD patients both at baseline and one year later.** For further explanations, see text.
(DOCX)

Information S1 **Members of the ECLIPSE Steering and Scientific Committees.** ECLIPSE Study Investigators and Study Centre Locations.
(DOCX)
