# Supplementary figures and images for: Persistent Systemic Inflammation is Associated with Poor Clinical Outcomes in COPD: A Novel Phenotype
Source: PLoS One. 2012 May 18;7(5):e37483. doi: 10.1371/journal.pone.0037483 (PMC3356313; doi:10.1371/journal.pone.0037483)

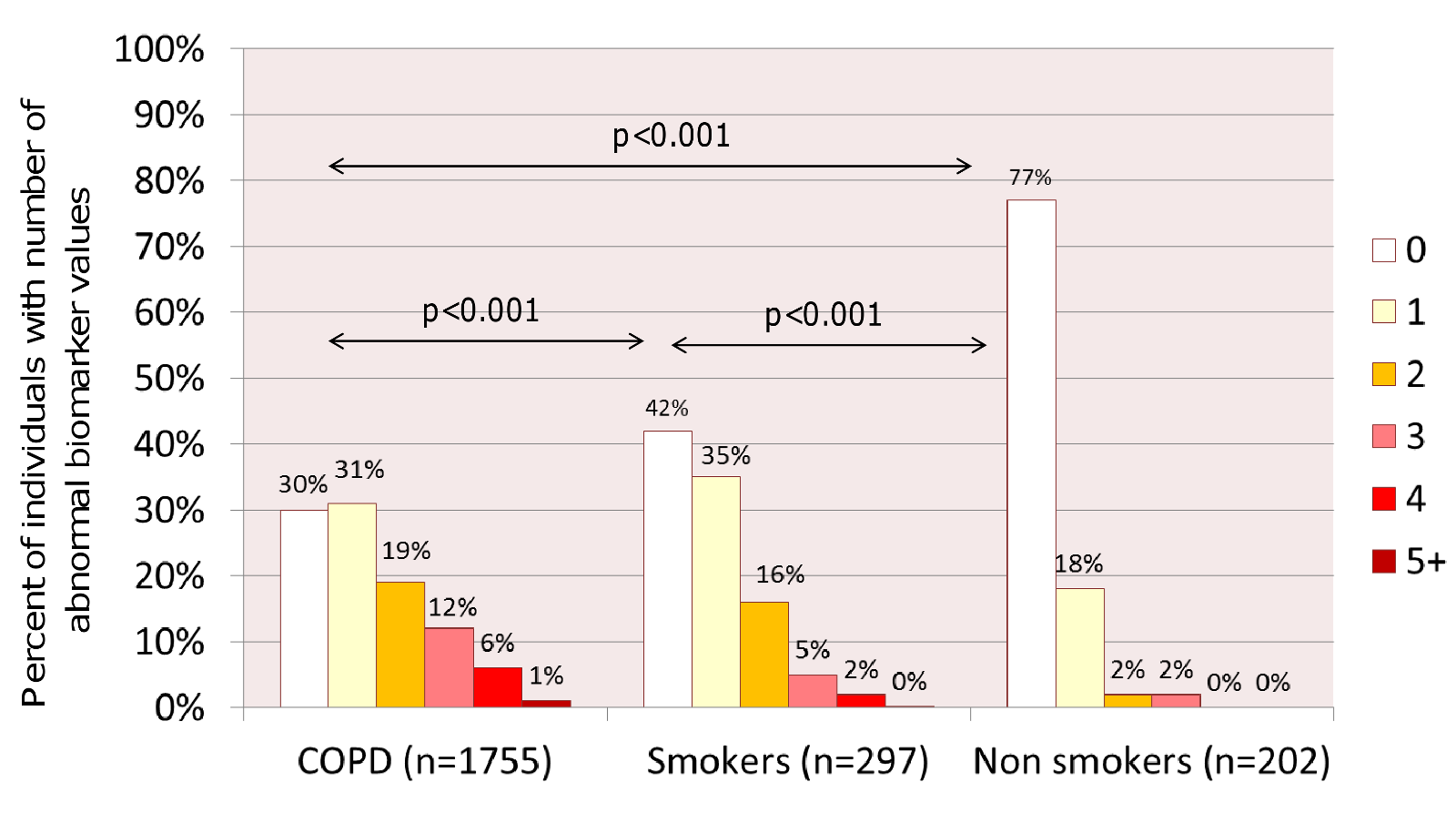

Supplement: Figure S1 — Frequency distribution of the percentage of individuals in each group with none, one or more abnormal biomarker values (>95th percentile of the nonsmoker controls) at baseline. For further explanations, see text. (TIF) [file pone.0037483.s001.tif]

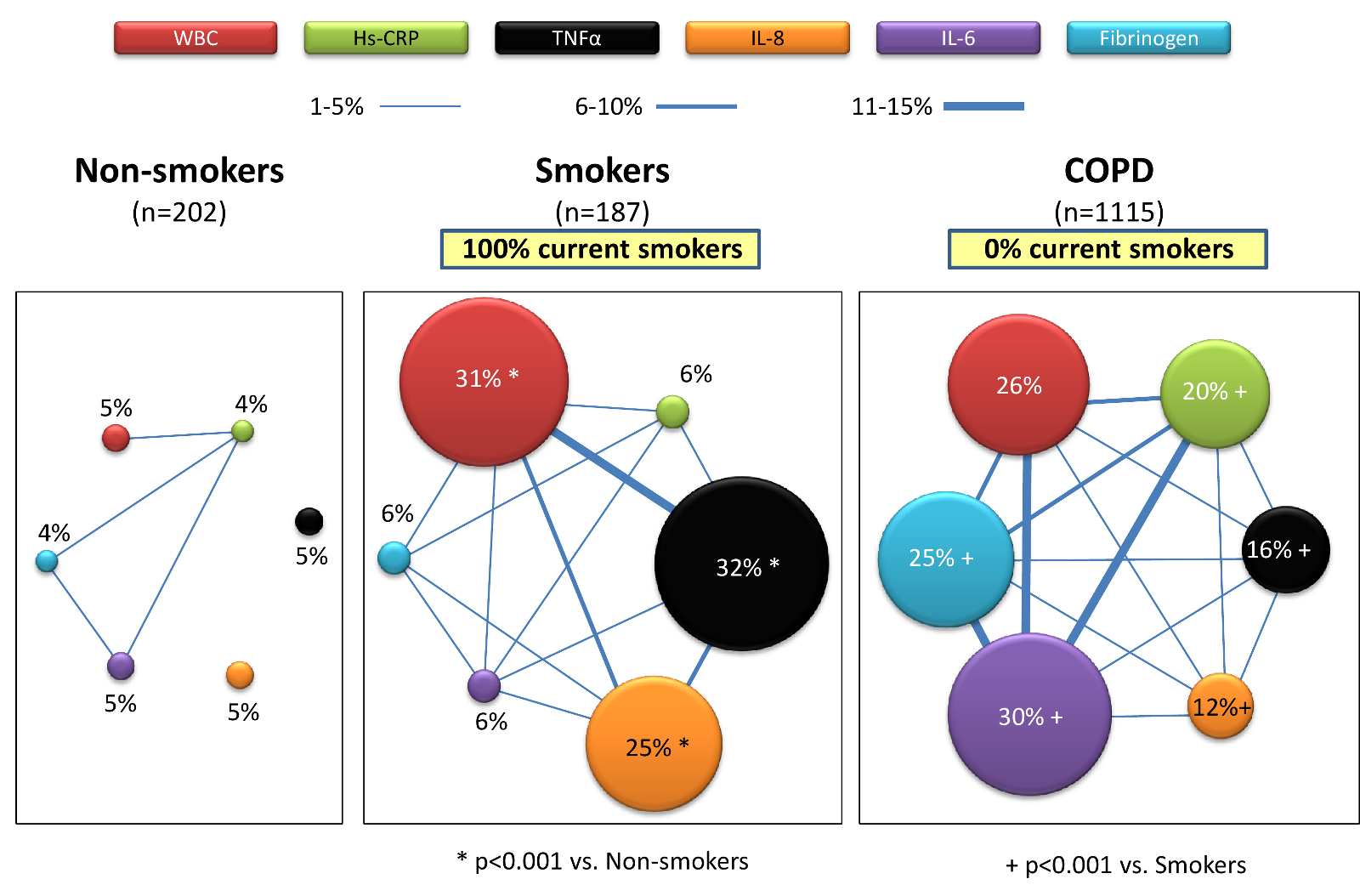

Supplement: Figure S2 — Systemic inflammome of non-smokers (n = 202), current smokers (only) with normal lung function (n = 187) and former-smokers (only) with COPD (n = 1115) at baseline. IL-8 and TNFα are very much influenced by current smoking whereas hs-CRP, IL-6 and fibrinogen are COPD-related inflammatory biomarkers. WBC counts are influenced both by smoking and COPD. For further explanations, see text. (TIF) [file pone.0037483.s002.tif]

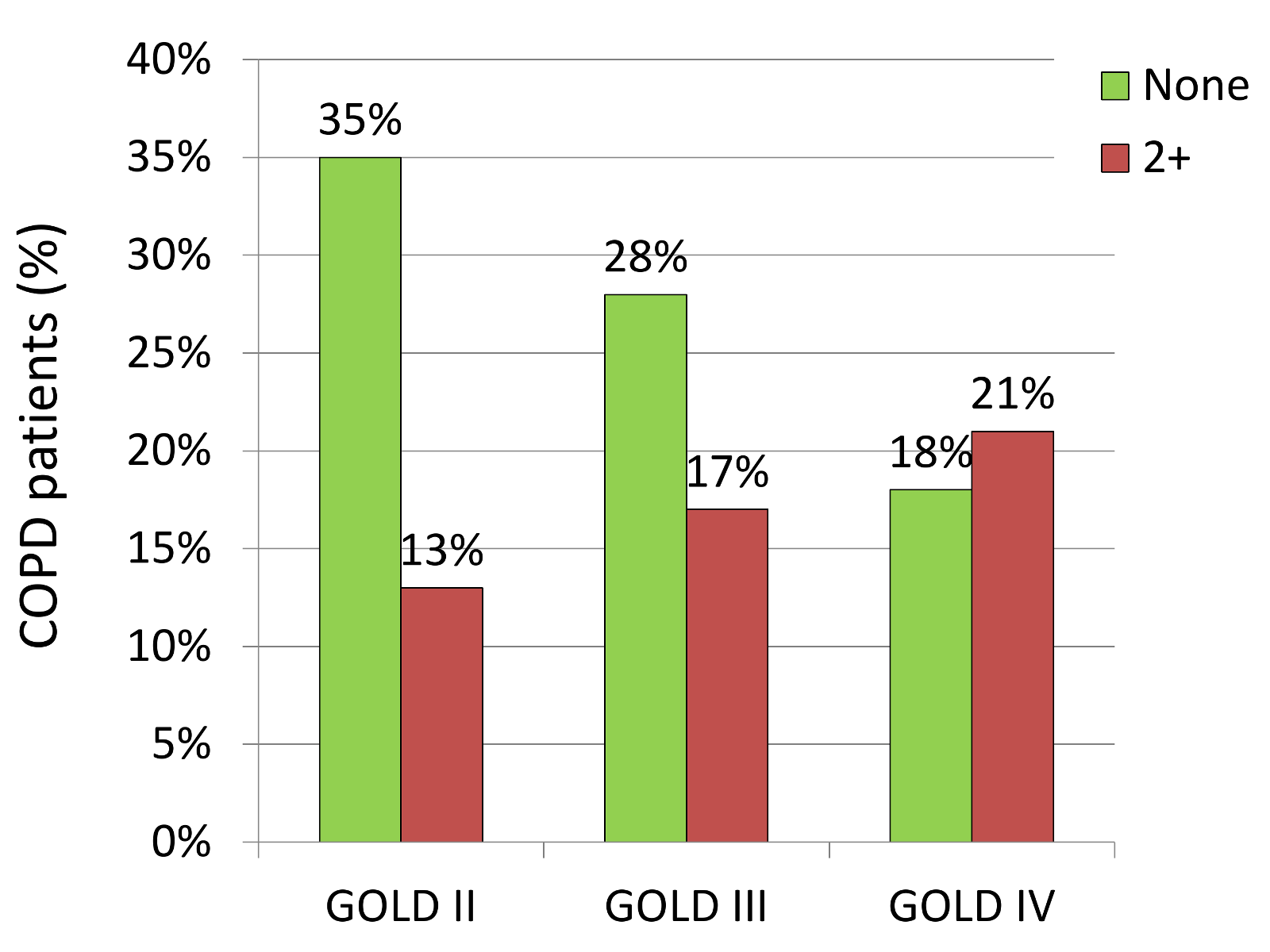

Supplement: Figure S3 — Percentage of COPD patients, by GOLD stage of airflow limitation severity, with none (blue bars) or 2+ biomarkers (red bars) in the upper quartile of the COPD distribution of values both at baseline and after one year follow-up. For further discussion, see text. (TIF) [file pone.0037483.s003.tif]

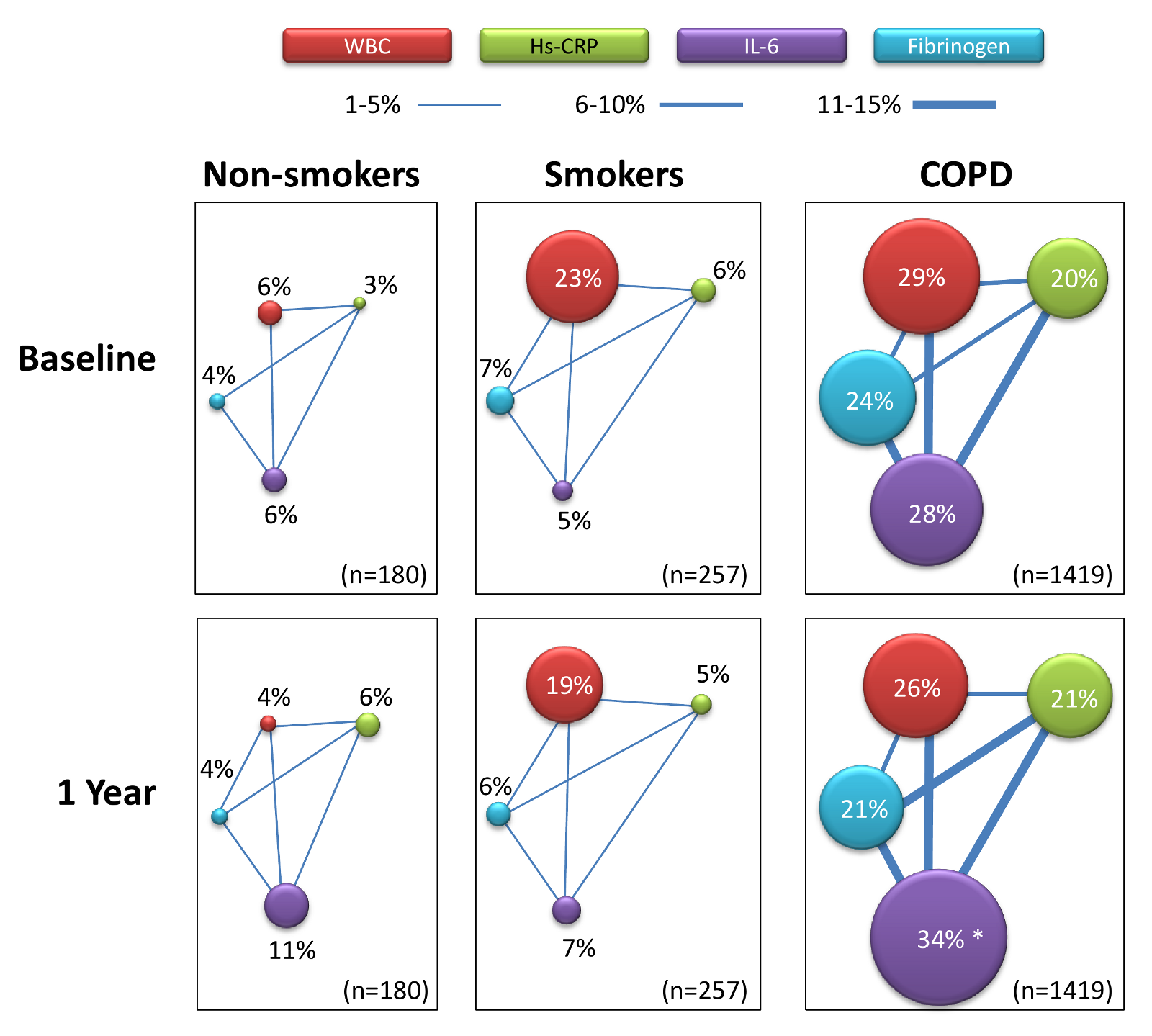

Supplement: Figure S4 — Systemic inflammome of the four biomarkers analyzed at baseline (upper panels) and at one year follow-up (bottom panels) in the same individuals in each group (note the same n value). Differences between groups were maintained after one year follow-up but were basically non-existent within groups, indicating stability of the systemic inflammome in each group. For further explanations, see text. (TIF) [file pone.0037483.s004.tif]
