# Supplementary material for: Persistent Systemic Inflammation is Associated with Poor Clinical Outcomes in COPD: A Novel Phenotype
Source: PLoS One. 2012 May 18;7(5):e37483. doi: 10.1371/journal.pone.0037483 (PMC3356313; doi:10.1371/journal.pone.0037483)
Supplement: Table S1 — Median [IQR] of the inflammatory biomarkers determined at baseline in COPD patients and smokers with normal lung function by smoking status. (DOCX) [file pone.0037483.s005.docx]

**Persistent Systemic Inflammation is Associated with Poor Clinical Outcomes in COPD: A Novel Phenotype**

Agustí et al.

**Table S1.** Median [IQR] of the inflammatory biomarkers determined at baseline in COPD patients and smokers with normal lung function by smoking status.

|  | **COPD** | | | **Smokers** | | |
| --- | --- | --- | --- | --- | --- | --- |
|  | **Current smoker**  **(n=640)** | **Former smoker**  **(n=1115)** | **p-value** | **Current smoker**  **(n=187)** | **Former smoker**  **(n=110)** | **p-value** |
| White Blood Cells (x10^6/ml) | 8.0 [2.6] | 7.3 [2.6] | <0.001 | 7.4 [2.7] | 6.6 [2.0] | <0.001 |
| High Sensitivity CRP (mg/l) | 3.1 [6.0] | 3.3 [5.4] | NS | 1.6 [2.2] | 1.6 [3.1] | NS |
| IL-6 (pg/ml) | 1.5 [2.3] | 1.5 [2.4] | NS | 0.6 [0.9] | 0.6 [1.0] | NS |
| IL-8 (pg/ml) | 7.3 [10.9] | 6.8 [9.3] | 0.095 | 9.1 [16.3] | 5.7 [7.7] | <0.001 |
| Fibrinogen (mg/dl) | 442.0 [131.5] | 450.5 [128.0] | NS | 387.0 [91.0] | 394.0 [82.0] | NS |
| TNF-α (pg/ml) | 2.4 [19.4] | 2.4 [3.0] | 0.004 | 17.2 [43.1] | 2.4 [29.7] | 0.005 |

NS: non-significant
