# Supplementary material for: Persistent Systemic Inflammation is Associated with Poor Clinical Outcomes in COPD: A Novel Phenotype
Source: PLoS One. 2012 May 18;7(5):e37483. doi: 10.1371/journal.pone.0037483 (PMC3356313; doi:10.1371/journal.pone.0037483)
Supplement: Table S2 — Median [IQR] of the inflammatory biomarkers determined at baseline in COPD patients by GOLD stages of airflow limitation. (DOCX) [file pone.0037483.s006.docx]

**Persistent Systemic Inflammation is Associated with Poor Clinical Outcomes in COPD: A Novel Phenotype**

Agustí et al.

**Table S2**. Median [IQR] of the inflammatory biomarkers determined at baseline in COPD patients by GOLD stages of airflow limitation.

|  | | | | **P-values** | | | |
| --- | --- | --- | --- | --- | --- | --- | --- |
|  | **GOLD II**  **(N=765)** | **GOLD III**  **(N=758)** | **GOLD IV**  **(N=231)** | **Overall** | **GOLD II vs GOLD III** | **GOLD II vs GOLD IV** | **GOLD III vsGOlD IV** |
| White Blood Cells (x10^6^/ml) | 7.4 [2.7] | 7.7 [2.5] | 8.1 [3.2] | 0.01 | NS | 0.003 | NS |
| hs CRP (mg/l) | 2.8 [4.8] | 3.5 [6.2] | 3.8 [7.4] | 0.02 | 0.01 | NS | NS |
| IL-6 (pg/ml) | 1.4 [2.1] | 1.6 [2.4] | 1.8 [3.6] | <0.001 | 0.01 | 0.001 | NS |
| IL-8 (pg/ml) | 7.4 [9.8] | 6.8 [10.3] | 6.0 [9.3] | 0.04 | 0.04 | 0.03 | NS |
| Fibrinogen (mg/dl) | 430.0 [117.0] | 456.0 [135.0] | 486.0 [147.0] | <0.001 | <0.001 | <0.001 | <0.001 |
| TNF-α (pg/ml) | 2.4 [25.9] | 2.4 [0.0] | 2.4 [3.8] | <0.001 | <0.001 | NS | NS |

NS: non-significant
