# Supplementary material for: Persistent Systemic Inflammation is Associated with Poor Clinical Outcomes in COPD: A Novel Phenotype
Source: PLoS One. 2012 May 18;7(5):e37483. doi: 10.1371/journal.pone.0037483 (PMC3356313; doi:10.1371/journal.pone.0037483)
Supplement: Table S3 — 95th percentile values of the six biomarkers determined in healthy non-smokers at baseline. For further explanations, see text. (DOCX) [file pone.0037483.s007.docx]

**Persistent Systemic Inflammation is Associated with Poor Clinical Outcomes in COPD: A Novel Phenotype**

Agustí et al.

**Table S3**. 95^th^ percentile values of the six biomarkers determined in healthy non-smokers at baseline. For further explanations, see text.

| **Biomarker** | **95^th^ percentile value** |
| --- | --- |
| White Blood Cells (x 10^6^/ml) | 8.6 |
| High Sensitivity CRP (mg/l) | 8.7 |
| IL-6 (pg/ml) | 2.6 |
| IL8 (pg/ml) | 20.8 |
| Fibrinogen (mg/dl) | 518.0 |
| TNFα (pg/ml) | 37.8 |
