# Supplementary material for: Persistent Systemic Inflammation is Associated with Poor Clinical Outcomes in COPD: A Novel Phenotype
Source: PLoS One. 2012 May 18;7(5):e37483. doi: 10.1371/journal.pone.0037483 (PMC3356313; doi:10.1371/journal.pone.0037483)
Supplement: Table S4 — Summary of 75th percentile value of the four biomarkers determined in COPD patients both at baseline and one year later. For further explanations, see text. (DOCX) [file pone.0037483.s008.docx]

**Persistent Systemic Inflammation is Associated with Poor Clinical Outcomes in COPD: A Novel Phenotype**

Agustí et al.

**Table S4**. Summary of 75^th^ percentile value of the four biomarkers determined in COPD patients both at baseline and one year later. For further explanations, see text.

| **Biomarker** | **Baseline** | **Year 1** |
| --- | --- | --- |
| White Blood Cells (x 10^6^/ml) | 8.9 | 8.8 |
| High Sensitivity CRP (mg/l) | 6.8 | 7.4 |
| IL-6 (pg/ml) | 2.9 | 3.4 |
| Fibrinogen (mg/dl) | 516.0 | 504.0 |
