# Supplementary material for: Persistent Systemic Inflammation is Associated with Poor Clinical Outcomes in COPD: A Novel Phenotype
Source: PLoS One. 2012 May 18;7(5):e37483. doi: 10.1371/journal.pone.0037483 (PMC3356313; doi:10.1371/journal.pone.0037483)
Supplement: Information S1 — Members of the ECLIPSE Steering and Scientific Committees. ECLIPSE Study Investigators and Study Centre Locations. (DOCX) [file pone.0037483.s009.docx]

**Supporting Information S1**

**ECLIPSE Steering and Scientific Committees (authors of this paper are shown in bold text); ECLIPSE Investigators (authors of this paper are shown in bold text) and Affiliations; ECLIPSE Study Centre Locations**

*ECLIPSE Steering Committee*: **Harvey Coxson** (Canada), **Courtney Crim** (GlaxoSmithKline, USA ), **Lisa Edwards** (GlaxoSmithKline, USA), **David Lomas** (UK), **William MacNee** (UK), **Edwin Silverman** (USA), **Ruth Tal-Singer** (Co-chair, GlaxoSmithKline, USA), **Jørgen Vestbo** (Co-chair, Denmark), **Julie Yates** (GlaxoSmithKline, USA).

*ECLIPSE Scientific Committee*: **Alvar Agusti** (Spain), **Per Bakke** (Norway), **Peter Calverley** (UK), **Bartolome Celli** (USA), **Courtney Crim** (GlaxoSmithKline, USA), **Bruce Miller** (GlaxoSmithKline, UK), **William MacNee** (Chair, UK), **Stephen Rennard** (USA), **Ruth Tal-Singer** (GlaxoSmithKline, USA), **Emiel Wouters** (The Netherlands).

*ECLIPSE Investigators*:

*Bulgaria*: Yavor Ivanov, Asthma Center, Pleven; Kosta Kostov, Military Medical Academy, Department of Pulmonology, Sofia.

*Canada*: Jean Bourbeau, Montreal Chest Institute, Montreal, Que; Mark Fitzgerald, UBC - Respiratory Medicine, Vancouver, BC; Paul Hernandez, Queen Elizabeth II Health Sciences Center, Halifax, NS; Kieran Killian, McMaster University, Health Sciences Center, Hamilton, On; Robert Levy, Pacific Lung Health Center, Vancouver, BC; Francois Maltais, Centre de recherche Hospital Laval, Montreal, Que; Denis O’Donnell, Kingston General Hospital, Kingston, On.

*Czech Republic*: Jan Krepelka, SPliN s.r.o, Praha.

*Denmark*: **Jørgen Vestbo**, H:S Hvidovre Hospital, Hvidovre.

*Netherlands*: **Emiel Wouters**, University of Maastricht, Maastricht.

*New Zealand*: Dean Quinn, Clinical Research, Wellington.

*Norway*: **Per Bakke**, University of Bergen, Bergen.

*Slovenia*: Mitja Kosnik, KOPA Golnik, Golnik.

*Spain*: **Alvar Agusti**, Thorax Institute, Hospital Clinic, IDIBAPS, University of Barcelona, and CIBER Enfermedades Respiratorias (CIBERES), FISIB, Mallorca.

*Ukraine*: Yuri Feschenko, Institute of Phthisiatry & Pulmonology, Kiev; Vladamir Gavrisyuk, Institute of Phthisiatry & Pulmonology, Kiev; Lyudmila Yashina, Institute of Phthisiatry & Pulmonology, Kiev; Nadezhda Monogarova, Donetsk State Medical University, Donetsk.

*United Kingdom*: **Peter Calverley**, University of Liverpool, Liverpool; **David Lomas**, University of Cambridge, Cambridge; **William MacNee**, University of Edinburgh, Edinburgh; David Singh, Medicines Evaluation Unit - Langley Bldg., Manchester; Jadwiga Wedzicha, The Royal Free Hospital, London.

*United States of America*: Antonio Anzueto, University of Texas Health Science Center San Antonio, TX; Sidney Braman, Rhode Island Hospital, Providence, RI; Richard Casaburi, Los Angeles Biomedical Research Institute at Harbor-UCLA Medical Center, Torrance CA; **Bart Celli**, Brigham and Women’s Hospital and Harvard Medical School, Boston, MA; Glenn Giessel, Pulmonary Associates of Richmond, Inc., Richmond, VA; Mark Gotfried, Pulmonary Associates, Phoenix, AZ; Gary Greenwald, Advances in Medicine, Rancho Mirage, CA; Nicola Hanania, Baylor Clinic, Houston, TX; Don Mahler, Dartmouth-Hitchcock Medical Center, Lebanon, NH; Barry Make, National Jewish Medical & Research Center, Denver, CO; **Stephen Rennard**, University of Nebraska Medical Center Omaha, NE; Carolyn Rochester, Yale University School of Medicine, New Haven, CT; **Paul Scanlon**, Mayo Clinic, Rochester, MN; Dan Schuller, Creighton University Medical Center, Omaha, NE; Frank Sciurba, University of Pittsburgh Medical Center Emphysema Research Center, Pittsburgh, PA; Amir Sharafkhaneh, Houston VA Medical Center, Houston, TX; Thomas Siler, Midwest Chest Consultants, St. Charles, MO, **Edwin Silverman**, Harvard University-Brigham & Women's Hospital, Boston, MA; Adam Wanner, University of Miami School of Medicine, Miami, FL; Robert Wise, Johns Hopkins Asthma & Allergy Center, Baltimore, MD; Richard ZuWallack, St. Francis Hospital & Medical Center, Hartford, CT.

**Appendix 2: Study Centre Locations**

| **Institution** | **Address 1** | **Address 2** | **City** | **State** | **Country** |
| --- | --- | --- | --- | --- | --- |
| University of Texas Health Science Center | Pulmonary Diseases | 7400 Merton Minter Blvd., (111E) | San Antonio | TX | USA |
| Rhode Island Hospital | Division of Pulmonary, Sleep & Critical Care Medicine | 593 Eddy Street APC 7th Floor | Providence | RI | USA |
| Los Angeles Biomedical Research Institute at Harbor-UCLA Medical Center | Rehab Clinical Trials Center | 1124 W. Carson St., Bldg J4 | Torrance | CA | USA |
| St. Elizabeth's Medical Center | Pulmonary STN - 3 | 736 Cambridge Street | Boston | MA | USA |
| Pulmonary Associates of Richmond, Inc. | 1000 Boulders Parkway | Suite 201 | Richmond | VA | USA |
| Pulmonary Associates, PA | 1112 East McDowell Road |  | Phoenix | AZ | USA |
| Advances in Medicine | 42362 Bob Hope Drive |  | Rancho Mirage | CA | USA |
| Baylor Clinic | 6620 Main Street | Suite 11B.16 | Houston | TX | USA |
| Dartmouth-Hitchcock Medical Center | Pulmonary & Critical Care Center | One Medical Center Drive | Lebanon | NH | USA |
| National Jewish Medical & Research Center | Weinberg Clinical Research Unit | 1400 Jackson Street | Denver | CO | USA |
| University of Nebraska Medical Center | Pulmonary Clinical Studies Unit | 982465 Nebraska Medical Center DRC II 1022 | Omaha | NE | USA |
| Yale University School of Medicine | Internal Medicine/Pulmonary | 1 Gilbert Street TAC S 441 | New Haven | CT | USA |
| Mayo Clinic | Pulmonary Clinical Research Center | Lanmark 2-46 14 - 2nd Street SW | Rochester | MN | USA |
| Creighton University Medical Center | Pulmonary & Critical Care Division | 601 N. 30th Street Suite 3820 | Omaha | NE | USA |
| University of Pittsburgh Medical Center Emphysema Research Center | 3471 5th Ave | Suite 1211 | Pittsburgh | PA | USA |
| Houston VA Medical Center | 2002 Holcombe Blvd. | Pulmonary 111-1 Room 3C-220 | Houston | TX | USA |
| Midwest Chest Consultants, PC | 330 First Capital Drive | Suite 470 | St. Charles | MO | USA |
| Harvard University-Brigham & Women's Hospital | Channing Laboratory | 181 Longwood Ave | Boston | MA | USA |
| University of Miami School of Medicine | 1600 NW 10th Ave | #7064-A (R-47) | Miami | FL | USA |
| Johns Hopkins Asthma & Allergy Center | 5501 Hopkins Bayview Circle | Room 3B-58 | Baltimore | MD | USA |
| St. Francis Hospital & Medical Center | Pulmonary Medicine | 114 Woodland Street | Hartford | CT | USA |
| Montreal Chest Institute | Respiratory Epidemiology and Clinical Research Unit | 3650 St Urbain  Rm K220 | Montreal | Quebec | Canada |
| UBC - Respiratory Medicine | 2775 Laurel St. | Room 7264 | Vancouver | British Columbia | Canada |
| Queen Elizabeth II Health Sciences Center | Infirmary Site, Rm 4464 | 1796 Summer Street | Halifax | Nova Scotia | Canada |
| McMaster University, Health Sciences Center | HSC 3U25 | 1200 Main Street W | Hamilton | Ontario | Canada |
| Pacific Lung Health Center | 8B Providence Wing | 1081 Burrard Street | Vancouver | British Columbia | Canada |
| Centre de recherche Hospital Laval | Pavillon U, Local 1751 | 2725 Chemin Sainte-Foy | Sainte-Foy | Quebec | Canada |
| Kingston General Hospital | Richardson House | 102 Stuart Street | Kingston | Ontario | Canada |
| Institut Clínic del Tòrax | Hospital Clínic, Universitat de Barcelona | Villarroel 170, Escala 3, Planta 5 | Barcelona |  | Spain |
| HAUKELAND UNIVERSITY HOSPITAL ECLIPSE Study | HAUKELANDSVN. 1 |  | Bergen |  | Norway |
| Aintree University Hospital | Respiratory Research Dept. | Longmoor Lane Ward 14A | Liverpool |  | UK |
| Asthma Center | Ivan Vazov str.31 | Pleven 5800 | Pleven |  | [Bulgaria](../../../../../../../../AppData/Local/Microsoft/AppData/Documents%20and%20Settings/rac40917/Local%20Settings/Temp/Documents%20and%20Settings/SVT61472/Local%20Settings/Temp/Local%20Settings/Temp/Local%20Settings/Temp/Local%20Settings/Temp/Local%20Settings/alg11089/Desktop/center_detail.asp) |
| KOPA Golnik | Golnik 36 | 4204 Golnik | Golnik |  | Slovenia |
| Military Medical Academy, Department of Pulmonology | 1, Sv Georrgi Sofiiski | Sofia 1606 | Sofia |  | Bulgaria |
| SPliN s.r.o | Cimicka 37/446 | 182 00Praha 8 | Praha |  | Czech Republic |
| Cambridge Institute of Medical Research | Wellcome Trust MRC bldg | Hills Road | Cambridge |  | UK |
| New Royal Infirmary Of Edinburgh | Little France | Old Dalkeith Road | Edinburgh |  | UK |
| P3 Research | Bown Hospital | Churchill Drive Crofton Downs | Wellington |  | NZ |
| Medicines Evaluation Unit - Langley Bldg. | Southmoor Road |  | Manchester |  | UK |
| H:S Hvidovre Hospital | Kettegaard Alle 30 |  | Hvidovre |  | Denmark |
| The Royal Free Hospital | Pond Street | Centre for Hepatology, Upper 3rd Floor | London |  | UK |
| Astmacentrum Hornerheide | Hornerheide 1 |  | Horn |  | Netherlands |
| Institute of Phthisiatry & Pulmonology | 10 Amosova str |  | Kiev |  | Ukraine |
| Institute of Phthisiatry & Pulmonology | 10 Amosova str |  | Kiev |  | Ukraine |
| Institute of Phthisiatry & Pulmonology | 10 Amosova str |  | Kiev |  | Ukraine |
| Donetsk State Medical University | 16 Prospekt Illicha |  | Donetsk |  | Ukraine |
